# Prevalence of Self-medication among MBBS Students of a Medical College in Kathmandu

**DOI:** 10.31729/jnma.4840

**Published:** 2020-02-29

**Authors:** Anjan Khadka, Kumud Kumar Kafle

**Affiliations:** 1 Department of Pharmacology, Nepalese Army Institute of Health Sciences (NAIHS), College of Medicine, Kathmandu, Nepal

**Keywords:** *drugs*, *medical students*, *practice*, *prescription*, *self-medication.*

## Abstract

**Introduction:**

Self-medication refers to self-prescription which includes diagnosing and treating one’s own illness and prescribing for one’s self. Though appropriate self-medication relieves acute symptoms, is time saving and economical, it should not be advocated because of more risks than benefits. Self-medication practices were found to vary in medical students of Nepal and India. This study was aimed to determine the prevalence of self-medication among medical students.

**Methods:**

This descriptive cross-sectional study was conducted among 76 MBBS students. The study involved the administration of the research questionnaire including demographic information, practice of self-medication, type of illness, factors influencing self-medication, commonly self-prescribed drugs, sources and strategies to reduce such practices. The data were analyzed using Graph pad prism version 6.

**Results:**

The prevalence of self-medication was 58 (76.6%), more common among first year students. The common illness found was headache and common drug self-prescribed was analgesic-antipyretic. The most common source of obtaining medicines for all three year medical students was pharmacy. Students were also prescribing medicines to family members, friends and juniors. More than half of the students 52 (68.4%) reported that self-medication practices should be encouraged.

**Conclusions:**

Self-medication had been widely practiced among medical students. Self-medication can relieve medical problems but also involve the risks of adverse drug reactions, resistance and masking of underlying diseases. Medical students should be given enough exposure for better understanding of rational prescribing to minimize self-medication. The further study on practice of self-medication is needed on various health professionals and even in general **community.**

## INTRODUCTION

Self-medication refers to self-prescription which includes diagnosing and treating one’s own illness and prescribing for one’s self.^1^ The World Health Organization (WHO) advocated that responsible self-medication can help in prevention and treatment of diseases that do not require medical consultation and provides a cheaper alternative for treating common ill-nesses.^2-4^

The MBBS curriculum of Institute of Medicine (IOM), Tribhuvan University, encourages self-directed, community and problem-based learning. College of Medicine-Nepalese Army Institute of Health Sciences (NAIHS) is affiliated to the IOM. The curriculum has been divided into three parts namely the first phase, the second phase and the third phase. Pharmacology is integrated in basic medical science (IBMS) during first phase (first and second year of MBBS).^5,6^ Self-medication practices was found to vary in medical students of different countries in previous studies.^3,6^ Similarly, in the absence of effective public health care system with sufficient supplies of essential drugs in developing countries, most people are forced to buy drugs from the private market.^7,8^

The aim of this study was to find the prevalence of self-medication among medical students in a medical college.

## METHODS

This descriptive cross-sectional study was conducted in College of Medicine, NAIHS. The study duration was 6 months from May 2018 to October 2018 after getting approval from Institutional Review Committee. Ethical approval was taken from Institutional Review Committee of NAIHS.

Convenience sampling was done and minimum sample size was calculated as,

n = z^2^ × (p × q)/e^2^

   = 1.64^2^ × (0.5 × 0.5)/0.1^2^

   = 67

Where,

n = minimum sample size
z = confidence interval at 90% z: 1.645
p = prevalence of self-medication taken as 50%
q = 1-p e= margin of error, 10%

The study was conducted among 76 students from first, second and final year MBBS. Students were selected according to their roll numbers printed in attendance sheet by convenience sampling method in the ratio 1.3:1.5:1 purposively (number of students in first, second and final year were 130, 150 and 100 respectively.

The inclusion criteria were students of first, second and final year present in the lecture hall willing to fill the questionnaire. The roll number was selected by multiple of 5 (i.e. roll numbers 5, 10, 15, etc.). Students unwilling to participate and those with roll numbers which are not multiple of 5 (i.e. roll number 1, 2, 3, 4, 6, 7, 8, 9, etc.) were excluded from the study. All selected students were segregated in lecture hall and requested to sit at gap of two chairs. They were made clear about details of questionnaire and asked for clarification of any confusion they had. The help of two third year students were taken to collect the questionnaires and to assure the filled data are mutually exclusive and not influenced by the other fellow participants. They were asked for their written consent in initial part of questionnaire and those who gave consent were provided with written as well as verbal instructions to fill up the questionnaire. The study involved the administration of the semi-structured questionnaires and the data generated on the measured characteristics are limited only to the specific period of the study. The data were collected in the form of filled questionnaire forms and the time given to fill up the form was 30 minutes. The questionnaire was in two parts. The first part contained questions on demographic information of the respondents such as age, gender, place of accommodation and year. Socio-economic variables such as health seeking behavior, names and sources of drugs used for self-medication, type of illness, factors influencing self-medication practices and strategies that may help reduce self-medication practices were covered in second part of questionnaire. The questionnaire consisted of close-ended and open-ended questions which were pre-tested for reliability and validity among 15 students of third year MBBS. A preformed questionnaire was distributed to the conveniently selected students of three batches at time of their respective self-directed learning hours i.e. on 3^rd^ and 4^th^ week of July.

The data were recorded in MS excel and analyzed using Graph pad prism version 6 and presented as simple descriptive statistics using percentages, numbers, tables and bar diagrams.

## RESULTS

The prevalence of self-medication among medical students was 58 (76.6%). The average age of the students from first year, second year and final year were 19.96±1.21, 20.8±1.15 and 24±0.85 years respectively. The minimum age was 18 years and maximum age was 26 years. The age-wise distribution of respondents is depicted (Table 1). Among 76 respondents, 42 (55.3%) were male and 34 (44.7%) were female. In first year (n=26), 16 (61.5%) were male and 10 (38.5%) were female, while in second year (n=30), 13 (43.3%) were male and 17 (56.7%) were female.

**Table 1:** Age of the respondents.

Age (Years)	Number of First year students	Number of second year students	Number of Final year students
18-20	18	12	0
21-23	8	18	6
24-26	0	0	14

Likewise in final year (n=20), 13 (65%) and 7 (35%) were male and female respectively (Table 2).

**Table 2:** Gender wise distribution of respondents.

Year	Male	Female
First Year	16	10
Second Year	13	17
Final Year	13	7
Total	42	34

Out of 76 students, 26 (34.2%) were living in their home with family, 36 (47.4%) were living in hostel and remaining 14 (18.4%) were living in rented rooms. None of the first year medical students were accommodated in hostel.

The number of respondents having some kind of illness during last two month of study period was 47 i.e. 22 (84.6%) from first year, 12 (40%) from second year and 13 (65%) from final year). The common illnesses present among students were headache 14 (29.8%), common cold and sore throat 11 (23.5%), fever 9 (19.1%) followed by diarrhea 7 (14.9%), allergy 6 (12.8%), acne 6 (12.8%), menstrual pain 4 (8.5%), nausea and vomiting 2 (4.3%) and dry eyes 1 (2%). The most common illness reported by first year and final year students was headache 6 (27.3% and 5 (38.5%) respectively), whereas second year students had common cold and sore throat 4 (33.3%).

The number of students practicing self-medication was 36 (76.6%) out of 47 students who had some kind of illnesses. Out of those practicing self-medication, the percentage of students from first year, second year and final year were found to be 18 (50%), 8 (22.2%) and 10 (27.8%) respectively. The common groups of drug that had been self-prescribed were analgesic-antipyretic 20 (56.5%), cough syrups/antitussives 12 (33.3%), antacids 9 (25%), antihistaminic 9 (25%) followed by antimicrobials 6 (16.7%), only by final year students, antispasmodics 5 (13.9%) for abdominal pain, and anti-diarrheal 3 (8.3%). The drug groups which were least used in self-medication were antiemetic (for nausea and vomiting), nasal decongestants (for common cold and nasal blockage) and eye drops (artificial tears) and these were used by final year students only.

Among 36 respondents who practiced self-medication, 21 (58.3%) students obtained their required medicines from private pharmacies, 8 (22.2%) students obtained from left over medicines from similar illness in the past and 7 (19.4%) students obtained from friends and relatives. The most common source of medicines for all three year medical students was found to be pharmacy.

**Figure 1:**
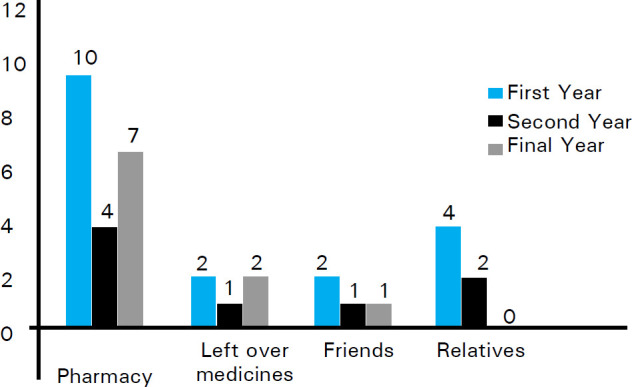
Sources of medicines.

The distributions of sources of drug among three years of students were compared (Figure 1).

Students had given multiple reasons for practicing self-medication i.e. previous experience of taking drugs for similar illness 24 (66.7%), fast relief from illness 9 (25%), not willing to visit doctors 8 (22.2%), exposure to drug advertisements 6 (16.7%), followed by non-serious nature of illness 5 (13.9%) and the knowledge about drugs acquired after joining MBBS 5 (13.9%). The most common reason for first year medical students for self-medication was non-serious nature of illness 7 (38.9%), whereas for second year and final year medical students, it was previous experience of taking drugs for similar illness 5 (62.5%) and 8 (80%) respectively.

**Figure 2:**
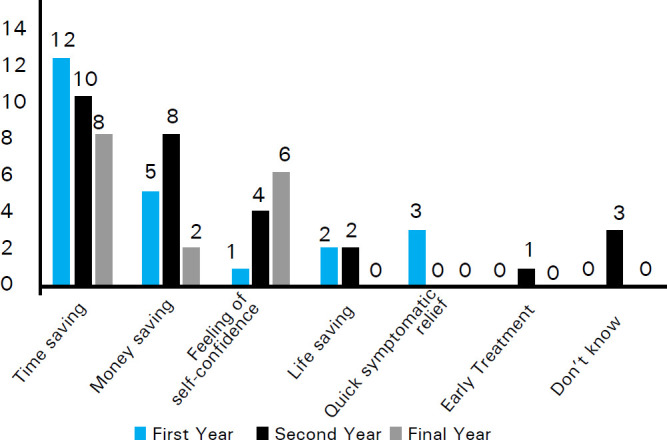
Benefits of self-medication.

**Figure 3:**
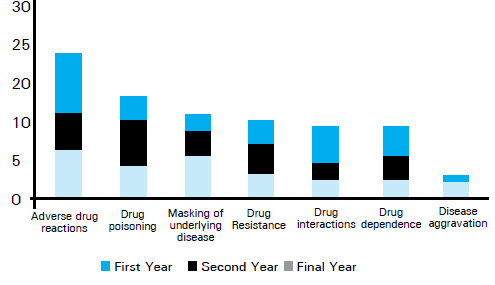
Harmful effects of self-medication.

Overall 52 (68.4%) students [12 (46.2%), 23 (76.7%) and 17 (85%) from first, second and final year respectively], thought that the self-medication practices should be encouraged as it is related with beneficial effects (Figure 2) whereas 24 (31.6%) students [14 (53.8%), 7 (23.3%) and 3 (15%) from first, second and final year respectively], thought that the self-medication practices should be discouraged as it is associated with harmful effects (Figure 3).

The common beneficial effects according to all three year students were time-saving with self-prescription 30 (57.7%) followed by money saving 15 (28.8%) and feeling of self-confidence 11 (21.2%). The 2 students each from first year (16.7%) and second year (8.75%) mentioned life-saving as the benefits of self-medication. Similarly, 3 (25%) first year students also mentioned prevention of complication as one of the benefits. Regarding the harmful effects of self-medication, the common harms as reported by students were adverse drug reaction 21 (87.5%) with the inadvertent use of drugs, drug interactions 17 (70.8%), masking of underlying diseases 10 (41.7%) and drug dependence 10 (41.7%). Other harms as cited by students were development of drug resistance 9 (37.5%), drug poisoning 4 (16.7%) and disease aggravation 4 (16.7%).

Out of total students involved in the study, 46% of them (16 from first year, 7 from second year and 12 from final year) advocated that the implications of self-medication among health care personnel and the consequences should be introduced to the syllabus of medicine at undergraduate level. However, 25% of students (8 from first year, 6 from second year and 5 from final year) thought that the easy availability and accessibility of medical facilities would be the effective way to minimize the self-medication practices among them. Only 8 (10.5%) students are in the favor of implementation of strict rules and regulations to reduce self-medication practices.

It was found that students were also involved in prescribing to others especially family members, friends and juniors (only by final year students). Out of total students involved in study, 28 (36.8%) students had prescribed drugs to others than self. The prescribing rate was higher among final year students 14 (70%) as compared to first year 5 (19.2%) and second year 9 (30%) medical students during last two months duration before commencement of study. Similarly, 11 (55%) final year students used to prescribe frequently

in comparison to 1 student each in first year (3.8%) and second year (3.3%). The common illnesses for which all three years students had prescribed were fever, diarrhea, headache, cough, dry eyes, whereas final year students had also prescribed in other illnesses like sore throat, oral ulcers, gastritis, hemorrhoids, dysmenorrhea, back pain, allergy, tonsillitis, pruritus and for vitamins supplementations.

## DISCUSSION

This study was focused among medical students of three different years of MBBS though they are not eligible to prescribe medicines. There are several studies on evaluation self-medication practices among medical students and professionals all around the world.^9-11^ This study showed that the prevalence of self-medication in those having illnesses was 76.6% (36 out of 47 students). Similar observations were made by Gyawali S et al. 227 (82.3%) among 276 students of second and fourth semester MBBS students in Nepal and Jagadeesh K et al. 66 (66%) among 100 second year medical students in India.^6,12^ The practice self-medication was found more in first year students followed by final year and second year students which is not in congruence with the previous studies that showed progressive increment in practice of self-medication as student progresses from first to final year.^13,14^ First year medical students had lesser knowledge regarding drugs and self-medication as compared to second year and final year students which made them vulnerable group. Second year students practiced relatively less because of their fresh knowledge of pharmacology. Final year students had practiced self-medication more than second year because they might be aware about responsible self-medication and the diseases during the clinical postings. Similarly, final year students might felt themselves in near to qualified prescriber to get prescription as compared to basic science students. Senior students also had appropriate and comfortably accessible alternatives to obtain medicine for self-medication.^14^ Our study revealed that the female students (48.57%) practiced self-medication more than male (30.95%) which is similar to the findings of Lukovic et al. which might be due to the perception of female towards drugs and their hesitancy in consulting doctors for the illness.^15-17^ Medical students tend to be curious and enthusiastic regarding drugs leading to the illegitimate prescription of medicine for themselves and others. Students attempt self-medication to get relief from the illness without the cost of seeing a doctor or sometimes as a result of fears associated with medical diagnosis as well as senior doctors. Our study showed that the students residing outside medical college hostel were practicing self-medication more as compared to those residing in college hostel. The exception with first year in our study could be due to lack of hostel facility for them. Students residing in hostel had medical infirmary room inside college premises where they can easily access medical doctors working as hostel wardens in both boy’s and girl’s hostel. They also had medicines free of cost. The students residing in home or rented rooms had no such facility and they had to access medical facility and drugs which incurs cost as well as time.

Our study observed that the most common group of drugs used in self-medication was analgesic-antipyretics. Similar results were observed by Lukovic et al. in their study among 1296 students from 1st, 3rd and 6th year students from School of Medicine University of Belgrade.^15^ In contrast to our study, Patil et al.reported antibiotics as most commonly self-medicated drugs in cross-sectional study among 440 undergraduate medical students of Karnataka, India.^17^ The final year students used more groups of drugs as compared to first and second year students. Analgesic-antipyretic drug like paracetamol was found to be the most commonly used over the counter drugs in our region.^4,13^ Students mostly self-medicated with over the counter drugs, however final year students also used antibiotics because of their knowledge regarding drugs and reluctance to visit doctor until and unless some severe presentation of disease or complications.

This study found that the major source of drugs for self-medication was private pharmacies followed by left over medicines, friends, family and relatives which are in congruence with previous study conducted in Nepal.^4^ There are many pharmacy shops in vicinity of college which have made easy accessibility and availability of drugs to students facilitating them to practice self-medication as mentioned in previous studies as well.^17,18^ In our region, people can easily purchase many drugs without prescription due to lack of stringent authority and lack of awareness regarding drug use, side effect, resistance and interactions as well as profit making attitude of some drug sellers.

The guidelines published by WHO for the regulatory assessment of medicinal products for use of self-medication listed education, family, society, law, availability of drugs and exposure to advertisements as influencing factors for practicing self-medication.^2^ The benefits of self-medication practices were found to be time-saving, money-saving, life-saving, feeling of self-confidence, quick symptomatic relief, prevent the complications and early initiation of treatment. First year and final year students said time saving is the most common benefits whereas second year students mentioned time and money saving as most common benefits. The results of study conducted in India and Egypt are in consistent with our results.^8,18,19^ Most of the first year students stated that the self-medication practices should be encouraged whereas only 23.33% of second year and 15% of final year students thought in similar way. The reason behind this difference might be better understanding of the self-medication in later years. However, around 30% of students are in the favor of discouraging self-medication. If self-medication is used widely without proper guidance, it might land with multiple risks such as drug resistance, tolerance, cross tolerance, hypersensitivity reactions, dependence, withdrawal symptoms, drug poisoning, etc.^20^ There is also a risk of using expired drugs, sharing them with friends or taking medicine that have been originally prescribed for some other problem. The students were aware about these hazards of self-medication. More number of first year students pointed out masking of underlying disease as the major drawback whereas second year students considered drug poisoning and final year students considered adverse drug reaction as major disadvantages of self-medication.

The students recommended to introduce self-medication in the syllabus of medicine at undergraduate level and also to provide medical facilities to all at ease and minimum cost so as to reduce practice of self-medication. Professional medical education might influence the perception of students towards self-medication practice and use of prescription only medicines. Medical students are not eligible to prescribe drugs to others unless and until they are qualified and registered to medical council but it was found that few students from first and second year, and 70% from final year are prescribing medicine to others in minor problems. This type of practices should be stopped and students should be discouraged to do so because this practice might relief symptoms momentarily but will mask the underlying disease and leads to disease complications as well as drug related problems.^20^

The limitation of this study included that the study involved lesser number of students and only from three batches of a single institute which might not be the representation for all medical students. The study was primarily based on self-reported data about self-medication in last six months so it makes difficult to avoid recall bias.

## CONCLUSIONS

Self-medication had been widely practiced among medical students. It was seen more in first year due to easy availability of drugs from pharmacy without prescription, little knowledge regarding drugs and hesitancy to visit doctors. The common illness found was headache and common drug self-prescribed was analgesic-antipyretic. The common source of self-prescribed drugs was private pharmacies. Self-medication is not devoid of risks of adverse drug reactions, toxicity, masking of underlying diseases and development of resistance. Medical students should be given enough exposure for the better understanding of rational practice of self-medication by incorporating the topic in their syllabus. More specific and larger scale study is required in multiple medical colleges and diverse health care settings as well as general population to evaluate the impact, role and burden of self-medication in our country.
